# Chronicles of the Octopus

**Published:** 1909-12

**Authors:** 


### CHRONICLES OF THE OCTOPUS.

### EVOLUTION OF THE GREAT MEDICAL TRUST.

It came to pass in those latter days that in the city called She Cargo (which being interpreted means "The Windy"), there dwelt a mighty man of ethics, whose name was Simon, surnamed The Boss. He was a wayfaring man from beyond the seas, aye, even from the country called Ireland (which being interpreted means the land of wrath), in the region round about Dublin, and was of the tribe of Hahneman-the-Homo. He blew into The Windy from the wilderness of Nebraska, even from the city of Lincoln, where he preached the gospel of Homeopathy and Water Cure for all the ills that flesh is heir to, from sore eyes to salpingitis, and proclaimed it from the housetops through a mighty trumpeter called *The Nebraska State Journal,* a secular sheet, in its Sunday editions.

Now it came to pass that in the year eighteen hundred and eighty-eight, or thereabout, this Apostle having waxed exceeding weak from such slim diet and the inability to gather much shekels, shook the dust of the wilderness off his feet, and, girding up his loins,—without purse or script,—journeyed unto the City of the Wind (and likewise of graft, trusts and combines), and there raised his Ebenezer.

Now this Simon took counsel with himself how best he could "raise the wind" and, incidentally, raise hell generally. He said: "Behold, am I not a versatile man, and can not I, like Paganini, play all tunes on one string? Can I not be anything that pays? Whether it be Homo, Osteo, Physio-Med or Regular? By the beard of Hahneman, the prophet, I'll do it. I'll be a Reformer for sure. For—behold, are the pathies not all our brothers; aye, verily, the Regulars (of the Tribe of Hippocrates), the Galenites and the Thompsonians (the disciples of The Herbes), the Osteos of the Tribe of Still-ites; aye, even the Ethiops of the Tribe of Sambo? These be all brothers, and I will gather them into the fold of the Octopus, and I shall reap much shekels. Moreover, are they all not divided on Therapeutics (which being translated means how best to bamboozle the public and gather coin)? And are not they—many of them, aye, even the best of them, gone astray, running after strange drugs called proprietaries, and tooting wind instruments—privately-owned-and-run-for-profit? Are they not hankering after the fleshpots of the Antis, and the 'Ines,' polluting the air and defiling the Temple of the A. M. A. with the breath of the Whore of Babylon, called Old Auntie Kome-nigh-us? Aye, verily. That same shall they not do." And he vowed a vow.

Straightway this great Simon summoned his principal adviser, one Priestly—a countryman of his—and took counsel of him, saying: "How shall I get control of that Monster Machine called The Octopus?" "Dead easy," says the High Priest(ly). "I'll put up a job on the Trustees. They shall fix the salary at such a figure that no American physician can accept the position of Boss (which is to give notice that no American need apply), and trust to luck to get a raise, later." And so it came to pass.

Now, being full-fed and flush, Simon waxed exceeding strong. He straightway called together his Council of Pharmacy (most of whom never saw a "pharm"), and he said: "Thus say I, The Boss: It shall not be lawful, or ethical, to advertise, dispense, sell, give away or prescribe any drug or combination not approved by my board. See to it. Give notice to the Scribes and Pharisees of the Tribe of Editors and the sect of Independents—so-called—that they shall not advertise any of the 'antis' or 'ines,' or trade-marked preps (unless the name be changed to conform to the U. S. P. and N. F. and N. A. R. P., because their substitutes, under Latin names are 'just as Gude,' but do not smell so good), and we will thus cut off the revenues of the run-for-profit heretics, who dare lift up their piping voices against *me*—even me, The Boss. And they shall perish from the earth forever. Selah!"

And lo, that all might hear the glad tidings of a newer ethics from the oracle and lawgiver of the Octopus, he summoned his Chief Spieler, one Mike Cor, of the Tribe of the Micks, and said: "Go thou into all the land, even unto Texas and the country roundabout Austin, and preach the gospel of Unification to all 'Docs' of whatever persuasion,—the Allos, the Homos, the Physio-Meds, the Eclectics, the Osteos (called also the Still-ites), aye, even to the Ethiops of the Tribe of Sambo, for behold! are they not our brothers?"

And the Chief Spieler, sometimes called The Walking Delegate, departed and journeyed into all the States, even unto Texas. He lifted up his voice in the Synagogues and in the county societies, and in the market places, crying aloud, "Organize! Organize! Round up the Docs! Corral them. Let none escape; no, not one, for he who sent me is greater than I, and he would have a great Trust, and get a corner on subscriptions for The Octopus. Every son of Aesculapius,—every son of Hahneman and of Still and of Galen and of Thompson,—every son of Ham of the Tribe of Sambo,—aye, every son-of-a-gun corraled, means five shekels a year, and they will get a copy of the great Octopus Organ weekly, whether they want it or not. Let none escape, under penalty of denunciation of being a scab and not in good standing." Thus saith Simon, The Boss.

And straightway Mac-the-Mick appointed captains of tens and captains of hundreds, called Councilors, and behold! the sects of Independents and their chief scribes—not seeing the intent—joined in with one voice and cried "Organize." And it came to pass that in each State there was a shaking up of dry bones, and every fellow hastened to enroll; for the fear of being a scab was heavy on them. Aye, the Independent-personally-owned-and-run-for-profit layouts all joined in the chase, and, like faithful collies, rounded up the "Docs" and drove them, like sheep to the shambles to be shorn; aye, like faithful deer hounds, they tracked the quarry for The Boss, who unheeding of the fate of Actæon did not foresee the day, now come, when those *hunting dogs* should turn upon and rend him.

And it came to pass that in addition to the captains of tens and captains of hundreds, called Councilors, there was appointed in each State a Captain of Hosts, who should be Chief Scribe and Keeper of the Records and Seal, and gather in the tribute. And every "Doc" in the land was made to stand and deliver, once a year, a tax of two shekels, and receive monthly a State Tooter, sometimes called a Tentacle, whether he wanted it or not. Now these Tooters were to echo the toots from the Tooter-in-Chief of ye Octopus, under penalty of going scalpless to bed. And the alacrity with which some of them obeyed was diverting. The old friends, the scribes of former years who had been faithful, efficient and loyal, were to be kicked out, and it was advertised that they were "unclean and infectious and unworthy of support."

And the Chief Spieler departed and again journeyed even unto Texas. He lifted up his voice in the Synagogues and the county societies and in the market places as before, and cried with a loud voice: "Repent ye, for the day of unification of all 'schools' and of mixed Boards is at hand." And certain ultra ethical disciples of Hippocrates and of Aesculapius,—aye, even of the Divine Apollo, who, in that ancient day (a few years ago) despised and reviled these sectarians, calling them quacks, and poked out their tongues at them in derision—fell over each other in their eagerness to embrace the Spieler.

And it came to pass that there was gathered together in the city called Atlantic, hard by the sea and nigh unto the shores of Jersey, a great multitude whose numbers wre as the sands of the shore. Black, white and tan,—Allo, Homeo, Osteo, Samb(e)o; and at the sound of the trumpet in the hands of the Chief Spieler—Mac-the-Mick, they all fell down and worshiped, saying, "Ain't he just the Real Thing?" and straightway The Boss caused a proclamation to be proclaimed, saying, "Be it known to all men that The Great Octopus, whose prophet I am (and there is none other), orders that no individually-owned-and-run-for-profit concern carrying unclean and infectious advertisements of the abomination class, called 'Antis,' shall be admitted to The Presence; for are they not unclean? Yea, every one. And are we not holier than they? We, and our Pharmacy Councilors and our Chief Tooters and Toadies? Selah. I have said."

Straightway all the scribes of the editorial sects of the Independents, when they heard these things, assembled together and took counsel, the one with the other. Much they marveled and were sore afraid. They lifted up their voices and wailed a big wail, that was heard from Dan to Beersheba,—the distance of a day's journey. "What manner of man is this?" said he of the *Cricket and Guy*. "On what meat doth he feed that he is grown so great? Is it not he who preached water cure, with homeopathy on the side, in the wilderness of Nebraska? Yea, verily! And did he not advertise in certain abominations called lay papers? Yea, in the Sunday editions thereof, after the manner of the quack and the charlatan? And is it he who now would instruct us, even us, the elect, in ethics? Go to. It shall not be. Have we not pulled the paps of dear Old Auntie Kam, lo, these many years, and waxed fat? Did she not give us suck when we were puling babes? And shall we now turn upon her and cast her into outer darkness?" And there was weeping and wailing and smashing of teeth.

But there were those amongst them who waxed bold and were exceeding wroth. These went unto their several sanctums scattered in the cities from the North to the South, from the East to the West,—even from Portsmouth to Charleston by the sea, and Savannah, and from New York to Texas, from the cities of the Middle West to the Gulf; and these doughty Knights of the Independent-run-for-profit-personally-owned concerns rashly fired dynamite cartridges at him—at long range—on the murderous toy pistol, so to speak, and even dared to shoot editorial squibs at him, and throw deadly spit balls at the Tyrant! They heaped anathemas on him. They reviled him and called him names. They wagged their heads and uttered cuss words. But, they all anted their yearly tithe of five shekels and *staid in,*—not even calling for a new deal.

Now it fell out that in the City of the Wind,—even of the She Cargo,—there dwelt certain Philistines of the tribe of The Live Wire and of the Square Deal. They took counsel of each other, chief amongst whom were G. Whiz Lydston, the Hard Hitter, and Abbott, surnamed the Strenuous, Chief of the Editorial Scribes and of the Square Dealers, and Robinovitch of the *Cricket and Guy.* These rose up and smote the Tyrant, hip and thigh, so that he was sore rattled; and he cried aloud to his man Friday (whose name is Jonesey, surnamed the Joke), Captain of the Hosts on the Golden Horn—even at the City of San, saying, "The Philistines be upon me, Jonesey, my Boy; whither shall I flee? Verily, they are after my scalp, and I am sore afraid."

And Jonesey answered him, saying, "Be not afraid, for lo, I am with you always. Know ye not that I am the same kind of a hairpin that you are? Have I not caught on, like you, after trying all things and holding fast to none? Have I not been, first and last, a jack of all trades, succeeding at none; a specialist for everything under the sun, but never till the day you discovered me have I struck pay gravel. I'm with you. Brace up."

And Simon was comforted; and they fell each upon the other's neck and wept tears. Yea, verily, they wept a great weep.

And it came to pass that when the King (no, the Boss, I mean) had assembled his wise men and his Council of Pharmacy and his Tooters of the several Tooting Organs that echoed the big toot, and his Rounder-up, Mac-the-Mick, and his host of "me-too" claquers, at a great feast in the City of the Wind, lo, there appeared a *handwriting on the wall,* and they were sore afraid and were seized with a great trembling.

And it came to pass that there was none there who could read it, for it was in an unknown tongue.

Now, be it remembered that at that time there dwelt in a far distant country—even in the land of the Long Horns and of the Secesh people, one Daniel, a prophet of great renown in interpreting dreams,—Daniel,—even he of the "Red Back." Him they sent for. And Daniel put on his specs, and, lifting up his voice, cried with a loud cry:

"MENE, MENE (Min-e-mo)

TEKEL (Me and I'll) TEKEL (You).

UPHARSIN!"

"Behold! *A House Divided Against Itself Must Fall. Thou Art Weighed in the Balance and Found Wanting.* Get thee back to the wilderness of Nebraska; yea, even unto thy Water Cure Institute; for we want thee not." Thus saith Daniel, the prophet. Selah!—*Texas Medical Journal*, January, 1909.

